# Interaction between genetic and epigenetic variation defines gene expression patterns at the asthma-associated locus 17q12-q21 in lymphoblastoid cell lines

**DOI:** 10.1007/s00439-012-1142-x

**Published:** 2012-01-24

**Authors:** Soizik Berlivet, Sanny Moussette, Manon Ouimet, Dominique J. Verlaan, Vonda Koka, Abeer Al Tuwaijri, Tony Kwan, Daniel Sinnett, Tomi Pastinen, Anna K. Naumova

**Affiliations:** 1 Department of Obstetrics and Gynecology, McGill University, Montreal, QC Canada; 2 The Research Institute of the McGill University Health Centre, Montreal, QC Canada; 3 Research Center, CHU Sainte-Justine, Montreal, QC Canada; 4 McGill University and Genome Quebec Innovation Centre, Montreal, QC Canada; 5 Department of Human Genetics, McGill University, Montreal, QC Canada; 6 Department of Pediatrics, University of Montreal, Montreal, QC Canada

## Abstract

Phenotypic variation results from variation in gene expression, which is modulated by genetic and/or epigenetic factors. To understand the molecular basis of human disease, interaction between genetic and epigenetic factors needs to be taken into account. The asthma-associated region 17q12-q21 harbors three genes, the zona pellucida binding protein 2 (*ZPBP2*), gasdermin B (*GSDMB)* and ORM1-like 3 (*ORMDL3*), that show allele-specific differences in expression levels in lymphoblastoid cell lines (LCLs) and CD4+ T cells. Here, we report a molecular dissection of allele-specific transcriptional regulation of the genes within the chromosomal region 17q12-q21 combining in vitro transfection, formaldehyde-assisted isolation of regulatory elements, chromatin immunoprecipitation and DNA methylation assays in LCLs. We found that a single nucleotide polymorphism rs4795397 influences the activity of *ZPBP2* promoter in vitro in an allele-dependent fashion, and also leads to nucleosome repositioning on the asthma-associated allele*.* However, variable methylation of exon 1 of *ZPBP2* masks the strong genetic effect on *ZPBP2* promoter activity in LCLs. In contrast, the *ORMDL3* promoter is fully unmethylated, which allows detection of genetic effects on its transcription. We conclude that the *cis*-regulatory effects on 17q12-q21 gene expression result from interaction between several regulatory polymorphisms and epigenetic factors within the *cis*-regulatory haplotype region.

**Electronic supplementary material:**

The online version of this article (doi:10.1007/s00439-012-1142-x) contains supplementary material, which is available to authorized users.

## Introduction

**Fig. 1:**
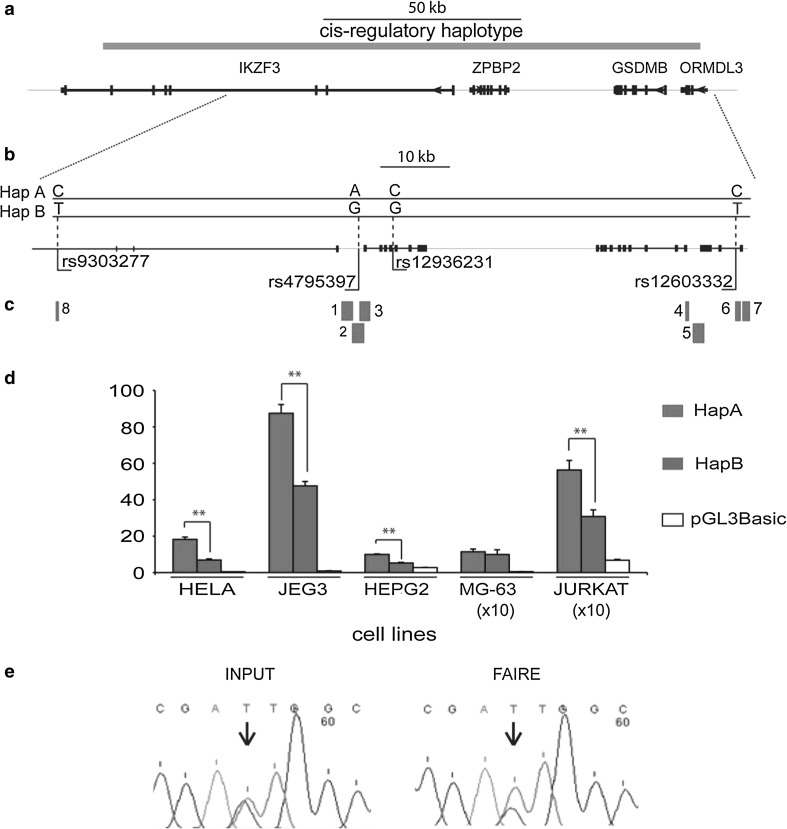
Functional analysis of the *cis*-regulatory region in 17q12-q21**. a** Genomic position of the *cis*-regulatory haplotype (hg18, chr17: 35,179,985-35,339,296) associated with allelic expression of *ORMDL3* and *GSDMB* (Verlaan et al. 2009a). **b** Positions of the common SNPs that form the *cis*-regulatory haplotype. For each SNP, the genotype associated with the haplotype A and B (HapA and HapB, respectively) is indicated on the *top*. HapA harbors the asthma-associated alleles and HapB harbors the non-asthma associated alleles. **c** Relative positions of the regions analyzed for in vitro promoter or enhancer activity. **d** Allelic differences in *ZPBP2* promoter activity in vitro. The *ZPBP2* promoter (region 2) that contains the rs4795397-A allele shows stronger promoter activity in vitro. A pGL3Basic plasmid has been used as negative control. The *Y*-*axis* indicates fold increase in transcription. Statistically significant allelic differences are indicated by *asterisks*; **e** allele-specific nucleosome occupancy detected by FAIRE at the rs4795397 region. Chromatograms for the input and FAIRE-enriched samples are shown. The position of the SNP rs4795397 is indicated by an *arrow*

Phenotypic variation is largely dependent on variation in gene expression levels. To identify the genetic determinants of phenotypic variation (including complex disease) in the human population, several genome-wide studies of genetically defined differences in gene expression levels succeeded to map *cis*-regulatory polymorphisms for a proportion of genes with variable expression (Dixon et al. 2007; Ge et al. 2009; Goring et al. 2007; Pastinen et al. 2004; Verlaan et al. 2009b; Yan et al. 2002). In a number of regions, including the chromosomal region 17q12-q21, genetic *cis*-effects act over several neighboring genes (Ge et al. 2009; Lluis et al. 2011; Verlaan et al. 2009a, b). Genome-wide association studies (GWAS) of gene expression in LCLs (Verlaan et al. 2009a, b) detected allele-specific differences in the expression of three genes: *zona pellucida* binding protein 2 (*ZPBP2),* ORM1-like 3 (*S. cerevisiae*) *(ORMDL3)* and gasdermin B (*GSDMB*) located in 17q12-q21 (Fig. 1a). This genomic interval is also associated with predisposition to early onset asthma, Crohn disease, ulcerative colitis and rheumatoid arthritis (Anderson et al. 2011; Barrett et al. 2008; Moffatt et al. 2007, 2011; Stahl et al. 2010). A *cis*-regulatory region responsible for the observed allele-specific differences in expression in CEPH LCLs has been mapped to a 160-kb long genomic interval that overlaps IKAROS family zinc finger 3 (*Aiolos*) (*IKZF3*), *ZPBP2*, *GSDMB* and *ORMDL3* (Verlaan et al. 2009a) (Fig. 1a). Two common *cis*-regulatory haplotypes, the asthma-associated HapA and the non-asthma associated HapB (also harboring the risk alleles for Crohn disease, ulcerative colitis and rheumatoid arthritis) have been delineated (Verlaan et al. 2009a) (Fig. 1a, b). HapA is associated with higher expression of *ORMDL3* and *GSDMB* and lower expression of *ZPBP2* whereas HapB is associated with an opposite pattern of gene expression, i.e. lower expression of *ORMDL3* and *GSDMB* and higher expression of *ZPBP2.* Expression of *IKZF3* is similar for both haplotypes (Verlaan et al. 2009a). Elucidation of the regulatory mechanism that underlies the effect of common polymorphisms on gene regulation is essential for the understanding of pathogenesis of asthma and other autoimmune diseases; therefore a search for functional *cis*-regulatory polymorphisms was undertaken. This search identified SNP rs12936231 that modifies a CTCF-binding site and influences nucleosome occupancy (Verlaan et al. 2009a). Suggestive functional results were found for several other SNPs from the candidate regulatory region. To further elucidate the transcriptional control of this asthma-associated locus, we focused on the interaction between genetic and epigenetic factors in the promoter regions of the three genes whose expression depends upon the *cis*-regulatory haplotype.

## Materials and Methods

### Cell culture of lymphoblastoid cell lines

HapMap LCLs were purchased from the Coriell Cell Repositories (Camden, NJ) and grown in T75 flasks in 1× RPMI 1640 Media (Invitrogen, Carlsbad, CA) (with 2 mM l-glutamine, 15% fetal bovine serum and 1% penicillin/streptomycin) at 37°C with 5% CO_2_. For formaldehyde-assisted isolation of regulatory elements (FAIRE) and chromatin immunoprecipitation (ChIP) assays, LCLs were grown to 90% confluence. Two independent cultures of cells were used for the FAIRE assay (input and FAIRE-treated cells).

### Transient transfection assays

To test for allelic activity, haplotype-specific constructs were subcloned into a pGL3 vector containing a firefly luciferase reporter gene either without a promoter or with an SV40 promoter (Promega, Madison, WI) using a previously published method (Belanger et al. 2005). All constructs were tested in five different human immortalized cell lines: cervical cancer (HeLa), choriocarcinoma (Jeg3), hepatocellular liver carcinoma (HepG2), osteosarcoma (MG-63) and CD4+ T-cell lymphoblast-like (Jurkat). These cell lines were transfected using lipofectamine^™^ 2000 according to the manufacturer’s protocol (Invitrogen, Carlsbad, CA). To control for transfection efficiency, the measurement of the firefly luciferase was normalized to the measurement of the *Renilla* luciferase. Experiments were performed in quadruplicate, the activities of the two luciferases were measured 24 h after transfection and allelic haplotypes for each SNP were compared. Statistical significance (*P* value) was determined using an unpaired Student’s *t* test.

### Formaldehyde-assisted isolation of regulatory elements (FAIRE) assays

The FAIRE procedure was performed as described (Giresi et al. 2007) with some modifications (Verlaan et al. 2009a). To test for FAIRE enrichment of specific SNP regions, 200–400 ng of DNA was amplified by PCR. For SNP regions that showed FAIRE enrichment, normalized Sanger sequencing was done. FAIRE-treated DNA samples were compared to the input DNA samples and normalized allelic ratios were calculated. The primers used for FAIRE analysis are listed in supplementary Table 2S.

### Chromatin immunoprecipitation (ChIP) assays

ChIP assays were performed as described in (Verlaan et al. 2009a). The following antibodies were used for ChIP assays: anti-histone H3K9Ac (06-942), anti-histone H3K27me3 (07-449); anti-C/EBP alpha (04-1104), anti-RNA Pol II (17-672) and anti-CTCF (07-729) (Millipore, Temecula, CA); anti-NFκB p65 (C-20), anti-YY1 (H-10) and anti-EP300 (C-20) (Santa Cruz Biotechnology, Inc.). Genomic regions known to be enriched for these proteins were used as positive controls [supplementary Table 2S and (Verlaan et al. 2009a)]. Promoter regions of the tumor necrosis factor receptor superfamily, member 1A (*TNFRSF1A*) and intercellular adhesion molecule 1 (*ICAM1*) genes that were used as positive controls for C/EBP alpha did not show enrichment, possibly due to antibody specificity. Primers used for quantitative PCR analysis or Sanger sequencing following ChIP assays are listed in the supplementary Table 2S.

### Sodium bisulfite sequencing methylation analysis

To establish the methylation patterns of regulatory regions, 0.5–2 μg of DNA was treated with sodium bisulfite as previously described (Clark et al. 1994) with modifications (Saferali et al. 2010). Assays were designed for each of the regions of interest. Nested PCR was performed for each of the loci. PCR products were purified using the MinElute gel extraction kit (Qiagen, Hilden, Germany) and cloned using the TOPO TA cloning kit (Invitrogen, Carlsbad, CA). The sequencing was done by the sequencing platform of the McGill University and Genome Quebec Innovation Centre. On average, 20 clones per sample were sequenced. Characteristics of regions, primers and PCR conditions are summarized in Supplementary Table 1S.

## Results

### Allelic differences in *ZPBP2* and *ORMDL3* promoter activity

To determine to what extent allelic differences in gene expression levels in the 17q12-q21 region were defined by genetic polymorphisms within gene promoters, the activity of annotated promoter regions of *ZPBP2*, *GSDMB* and *ORMDL3* was tested in in vitro transfection assays in five different cell types (Table 1; Fig. 1c). The annotated *GSDMB* promoter region did not show significant promoter activity in any of the cell types tested (region 4, Table 1). Two putative *ORMDL3* promoter regions were tested. The promoter region for the major *ORMDL3* isoform showed high promoter activity in all tested cell lines with no allelic effect (region 7, Table 1), whereas the putative promoter region for the minor isoform of *ORMDL3* (region 6) that included SNP rs12603332 (C/T) showed promoter activity in MG63 cells with a strong allelic effect. The construct that carried the haplotype HapA-associated rs12603332-C allele had higher promoter activity (*P* < 0.01, Student’s *t* test) (Table 1). However, exome sequencing data suggest that this promoter is not active in LCLs (Kwan et al. 2009). The construct including both promoters maintained high promoter activity; however, the allelic effect was lost (Table 1).

**Table 1:** Promoter activity of putative regulatory regions tested using in vitro transfection assays

Region	Annotated promoter region	Position (hg18)	Fragment size	Average fold increase of activity compared to basic pGL3 vector (range HapA-HapB)					Allelic effect
				Cell line					
				HELA	JEG3	HEPG2	MG63	JURKAT	
2	ZPBP2	chr17: 35,276,297–35,278,101	1,805 bp	28** (40.4–15.3)	79.5** (103–56)	2.8* (3.7–2)	22.4 (22.8–19.8)	6.5** (8.3–4.5)	Yes
3	ZPBP2	chr17: 35,277,475–35,279,042	1,568 bp	0	8.4* (9.5–7.3)	0	0	0	Yes
4	GSDMB	chr17: 35,328,506–35,329,058	552 bp	0	2.0	1.6	2.8	0	No
6	ORMDL3 isoform 2	chr17: 35,336,107–35,337,106	1,000 bp	0	3.0** (3.8–2.2)	3.5** (4.6–2.4)	14.7** (19.4–9.4)	0	Yes
7	ORMDL3 isoform 1	chr17: 35,337,322–35,338,541	1,220 bp	349	185	41	222	5.1	No
6 + 7	ORMDL3 isoforms 1 and 2	chr17: 35,336,107–35,338,541	2,434 bp	531	298	68	502	8	No

* Significant allelic effect *P* < 0.05

** Significant allelic effect *P* < 0.01

**Table 3:** Enhancer activity of putative regulatory regions tested using in vitro transfection assays

Region	Position with respect to genes	Position (hg18)	Fragment size	Average fold increase of activity compared to pGL3SV40 vector (range HapA-HapB)					Allelic effect
				Cell line					
				HELA	JEG3	HEPG2	MG63	JURKAT	
1	ZPBP2-promoter region	chr17: 35,274,877–35,276,528	1,652 bp	0	0	0	0	0	Not informative
2	ZPBP2-promoter region	chr17: 35,276,297–35,278,101	1,805 bp	1.4** (1.6–1.3)	8.6* (9.2–8.0)	0	2.1 (2.3–1.9)	1.3** (1.4–1.2)	Yes
3	ZPBP2-promoter region	chr17: 35,277,475–35,279,042	1,568 bp	0	4.8* (5.2–4.4)	0	1.4 (1.5–1.3)	0	Yes
5	ORMDL3 3’ region	chr17: 35,329,523–35,331,509	1,987 bp	0	0	0	4.0	0	No
6	ORMDL3 isoform 2 promoter region	chr17: 35,336,107–35,337,106	1,000 bp	0	1.4	0	3.0	0	No
7	ORMDL3 isoform 1 promoter region	chr17: 35,337,322–35,338,541	1,220 bp	3.8	6.5	2.0	7.8	0	No
8	Rs9303277	chr17: 35,229,745–35,230,240	495 bp	0	1.7	0	1.45	0	No

* Significant allelic effect *P* < 0.05

** Significant allelic effect *P* < 0.01

In the *ZPBP2* promoter region, the construct that carried the HapA-associated rs4795397-A allele in HeLa, Jeg3, HepG2 and Jurkat cells showed higher promoter activity (*P* < 0.01, Student’s *t* test) (Fig. 1d and region 2; Table 1). A partially overlapping construct that contained the transcriptional start site, exons 1 and 2 of *ZPBP2* was active only in JEG3 cells and showed a significant allelic effect (*P* < 0.05, Student’s *t* test) (region 3, Table 1).

In conclusion, the asthma-associated HapA haplotype variants of the *ZPBP2* promoter region and the putative promoter for the minor *ORMDL3* isoform had higher in vitro promoter activity compared to the variants associated with the HapB haplotype.

### Allele-specific regulatory elements

C*is*-regulatory allelic effects may arise from allele-specific differences in transcription factor binding and enhancer activity (Agueda et al. 2011; Bickel et al. 2011; Colombo et al. 2011; Harmon et al. 2010; Mertens et al. 2010). Transcription factor binding to a regulatory DNA element usually results in repositioning of nucleosomes. Allelic effects of putative regulatory SNPs on nucleosome positioning were explored using the FAIRE assay that identifies DNA regions with reduced nucleosome occupancy, i.e. regions potentially associated with transcription factors (Giresi et al. 2007). Two of the 22 tested SNP regions, rs12936231 and rs4795397, showed both an overall FAIRE enrichment and allelic differences in nucleosome occupancy (Verlaan et al. 2009a). The effect of SNP rs12936231 on nucleosome occupancy and CTCF-binding has been described in detail elsewhere (Verlaan et al. 2009a). The SNP rs4795397 residing in the proximal promoter region of *ZPBP2* also influenced FAIRE enrichment in five of six heterozygous LCLs tested. The rs4795397 A-allele had about twofold higher FAIRE enrichment than the rs4795397-G allele (Fig. 1e). The A-allele is part of the asthma-associated haplotype HapA and is associated with lower expression level of *ZPBP2* in CEU LCLs. However, it shows higher promoter activity in vitro (Fig. 1d). Hence, overall our data indicate that the allele that confers higher promoter activity in in vitro gene reporter assays and is associated with reduced nucleosome occupancy, i.e. with transcription factors in vivo*,* surprisingly, is the same allele that is associated with lower expression levels of the *ZPBP2* gene.

The Encode ChIP-sequencing results show enrichment of at least twelve transcription factors within the rs4795397 region (Myers et al. 2011; Raney et al. 2011). These include the nuclear factor of kappa light polypeptide gene enhancer in B-cells 1 (NFκB) p65 subunit, which is a central player in inflammation and immunity, RNA polymerase II (RNA POL II), and the transcriptional co-activator E1A binding protein p300 (EP300) (supplementary Fig. 1S). Analysis of the DNA sequence of the rs4795397 region (Transcription Element Search System database, http://www.cbil.upenn.edu/cgi-bin/tess) predicts binding sites for Yin and Yang 1 (YY1) and the CCAAT/enhancer binding protein (C/EBP) alpha transcription factors that overlap the SNP. To determine if SNP rs4795397 influences transcription factor binding in vivo, enrichment with NFκB, EP300, YY1, C/EBP alpha, RNA POL II and insulator protein CTCF was tested in LCLs that were homozygous for either the rs4795397-A or the rs4795397-G allele using ChIP. The region was enriched with NFκB, YY1, EP300 and RNA POL II (Table 2). High inter-individual variation between cell lines with respect to transcription factor enrichment and no statistically significant effect of the genotype were observed. We tested the allelic effect on NFκB alpha and RNA POL II enrichment using ChIP followed by Sanger sequencing in two heterozygous cell lines. No significant allelic differences in enrichment were detected (Table 2). We conclude that NFκB, RNA POL II, YY1 and EP300 bind both alleles in the rs4795397 region.

**Table 2:** Enrichment of the rs4795397 region chromatin with transcription factors in LCLs

ChIP	Enrichment			Allelic effect tested by Sanger sequencing in heterozygous LCLs (number of LCLs tested)
	All genotypes (number of LCLs tested)	Homozygous HapA (number of LCLs tested)	Homozygous HapB (number of LCLs tested)	
NFkB	2.06 ± 0.60 (8)	2.42 ± 0.73 (3)	1.57 ± 0.14 (3)	Absent (2)
CTCF	1.06 ± 0.46 (8)	1.11 ± 0.51 (4)	1.01 ± 0.49 (4)	nt
YY1	3.36 ± 1.57 (5)	3.58 ± 2.16 (3)	3.03 (2)	nt
EP300	2.63 ± 0.91 (4)	2.86 (2)	2.39 (2)	nt
RNA POL II	6.75 (2)	nt	nt	Absent (2)
Histone H3K9Ac	57.41 ± 20.95 (4)	50.44 (2)	64.27 (2)	nt
Histone H3K27me3	1.98 ± 0.20 (4)	1.92 (2)	2.04 (2)	nt

Standard deviation is given if three of more LCLs were tested

*nt* not tested

The rs4795397 region was also highly enriched for the active histone mark H3Ac, and showed low enrichment for the inactive histone mark H3K27me3 that were also independent from genotype (Table 2). C/EBP alpha ChIP results were not conclusive as enrichment was not detected in any of the regions tested including positive controls, perhaps due to antibody specificity.

The transcriptional control of genes within the 17q12-q21 chromosomal region is poorly understood and enhancers that regulate *ORMDL3* and *GSDMB* expression have not been yet identified. To locate putative enhancers, we searched the publically available data [UCSC database (Raney et al. 2011; Myers et al. 2011)] for genomic regions that were enriched for enhancer-specific epigenetic marks e.g. histones H3K4me1 and H3K27Ac; and/or the transcriptional co-activator E1A binding protein p300 (EP300) (supplementary Fig. 1S). These regions were tested for in vitro enhancer activity (Fig. 1; Table 3 and supplementary Fig. 1S). The candidate enhancer region overlapping with the 5′ region of the *ZPBP2* gene was too large and had to be tested as 3 separate overlapping constructs (regions 1–3 in Table 3). Enhancer activity was detected for the *ZPBP2* promoter region (region 2) in Jeg3 and MG63 cells, for region 1 in Jeg3 cells; for the *ORMDL3* promoter (region 6) and 3′ regions in MG63 cells; for the *ORMDL3* promoter (region 7) in all cell lines except Jurkat cells (Table 3). Significant allelic effects were observed for regions 2 and 3 (Table 3).

Collectively, our data demonstrate that the common SNP rs4795397 is a regulatory polymorphism that affects promoter activity, nucleosome positioning and is part of an enhancer region.

### DNA methylation of promoter regions

**Fig. 2:**
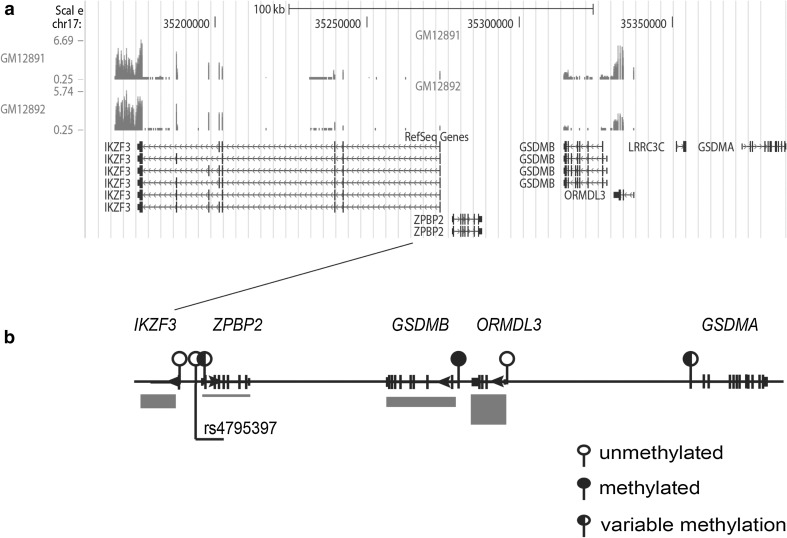
Promoter methylation and gene expression patterns in the 17q12-q21 region**. a** Exome sequencing results for the region 17q12-q21 in two LCLs (GM12891 and GM12892). **b** Summary of DNA methylation results for the 17q12-21 region. *Red rectangles below the diagram* reflect the relative RNA abundance for genes in the region

Monoallelic expression of certain X-linked and imprinted genes results from allelic differences in promoter methylation. To determine if promoter methylation had an effect on the expression of the 17q12-q21 genes in LCLs, methylation profiles of the annotated *IKZF3*, *ZPBP2*, *GSDMB,* *ORMDL3* and *GSDMA* promoters and first exons were determined (Fig. 2). The *ORMDL3* and *IKZF3* promoters were unmethylated in all tested cell lines independent from their genotypes (supplementary Figs. 2S, 3S). The annotated *GSDMB* promoter and exon 1 of isoform 2 were highly methylated in all genotypes [11 LCLs were tested, (supplementary Fig. 4S)] suggesting that transcription of the major annotated isoform 2 of *GSDMB* was suppressed in LCLs, which is in agreement with the exome sequencing data (Fig. 2a). It is worth noting, however, that the haplotype HapA contains polymorphisms that abolish three out of seven CG sites in the annotated *GSDMB* promoter. Moreover, this region had slightly lower mean methylation levels in LCLs that were homozygous for the HapA haplotype (*n* = 3; mean methylation level 79.7%) compared to LCLs that were heterozygous (*n* = 4, mean methylation level 95.7%) or homozygous for the HapB haplotype (*n* = 4, mean methylation level 95.6%). For all 11 LCLs, the methylation level of the *GSDMB* promoter and exon 1 was inversely correlated with RNA abundance (Pearson’s correlation coefficient *r* = −0.63, α = 0.05).

**Fig. 3:**
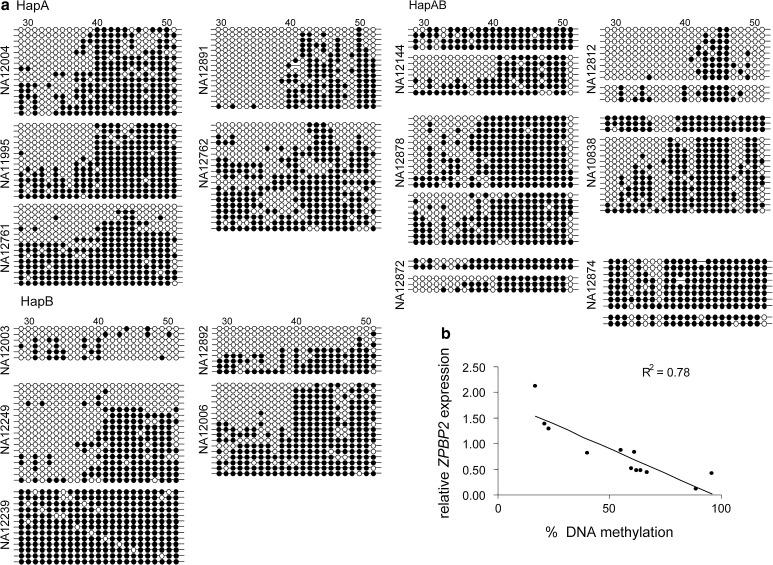
Variable DNA methylation of *ZPBP2* exon 1 defines *ZPBP2* expression levels**. a** *Filled circles* represent methylated cytosines, *open circles* represent unmethylated cytosines in CG pairs. *Each row* represents the methylation pattern of a single clone, i.e. one allele. The CG ID number is shown on the *top of the panel* and the ID of the cell line is shown on *the left*. A total of 51 CG sites were analyzed. CGs 29-51 that are located within exon 1 of *ZPBP2* and have greater variability in DNA methylation are shown in the figure. **b** Negative correlation between *ZPBP2* exon 1 methylation and *ZPBP2* RNA abundance. *ZPBP2* expression was evaluated using real-time RT-PCR and normalized to the 18S RNA levels as described in (Verlaan et al. 2009a)

In contrast to *ORMDL3* and *GSDMB* promoters, the *ZPBP2* promoter and exon 1 region showed highly variable DNA methylation patterns both within and between cell lines (Fig. 3a). Sixteen LCLs were tested. The *ZPBP2* promoter was highly methylated in cell lines homozygous for the asthma-associated HapA haplotype (*n* = 5) and in heterozygous cell lines (*n* = 6), but had lower methylation levels in cell lines that were homozygous for the non-asthma associated HapB haplotype (*n* = 5). To determine if *ZPBP2* methylation depended upon the parental origin of the allele, we compared the methylation profiles of maternal and paternal alleles in two LCL DNA samples, NA10838 and NA12878. No significant parental origin effect was detected.

Comparison of *ZPBP2* promoter and exon 1 methylation and expression levels showed a strong inverse correlation between methylation of exon 1 and *ZPBP2* RNA levels (Pearson’s correlation coefficient *R* = −0.88, α = 0.05) (Fig. 3b). Hence, methylation levels of *ZPBP2* exon 1 influence *ZPBP2* RNA levels and explain the apparent contradiction between high in vitro activity and FAIRE enrichment of the *ZPBP2* promoter region and lower expression of *ZPBP2* in LCLs that carry the asthma-associated haplotype HapA.

*GSDMA* is not expressed in LCLs (Fig. 2a), therefore LCLs are not the appropriate model for testing genetic *cis*-regulatory effects on its expression. However, increased expression of *GSDMA* was found in cord blood lymphocytes of individuals that carry the asthma-associated 17q12-q21 alleles (Lluis et al. 2011), suggesting that *GSDMA* cannot be excluded from the list of putative asthma genes. Hence, to obtain a complete picture of promoter methylation in the 17q12-q21 region we determined the methylation profile of the *GSDMA* promoter region in LCLs and found inter-individual variation among LCLs with respect to methylation levels (supplementary Fig. 5S).

We also tested the methylation profile of the rs4795397 region for allelic effects and found that it was unmethylated independent of genotype (supplementary Fig. 6S).

Overall, the methylation profiles of promoter regions show a good correlation with the expression levels of respective genes, i.e. highly expressed transcripts such as *IKZF3* and *ORMDL3* have completely unmethylated promoters, while genes with even partial promoter methylation show a considerably reduced transcriptional activity.

## Discussion

The asthma-associated chromosomal region 17q12-q21 harbors several genes that show allelic differences in expression in LCL. Our data suggest that allelic variation in expression arises from the interaction between several genetic polymorphisms and epigenetic factors. We have previously reported the effect of the common SNP rs12936231 on CTCF binding and nucleosome occupancy (Verlaan et al. 2009a). In the present study, we demonstrate that another common SNP, rs4795397 that is part of the *cis*-regulatory haplotype and is located within the promoter region of the *ZPBP2* is a putative functional polymorphism that shows allele-specific nucleosome occupancy and in vitro promoter activity. The rs4795397 region is enriched with YY1 and co-activator protein EP300. YY1 and EP300 are known to form regulatory complexes that may repress (Galvin and Shi 1997; Lee et al. 1995) or activate (Mokrani et al. 2006, Baumeister et al. 2005) gene transcription in response to different stimuli including endoplasmic reticulum stress and viral infection. The rs4795397 region is enriched with the active histone mark H3K9Ac, but not the repressive chromatin mark H3K27me3, an observation which is consistent with the histone acetyltransferase activity of EP300 (Ogryzko et al. 1996). Overall, the ChIP and FAIRE results indicate an active chromatin state at the rs4795397 region. Furthermore, our data show that although rs4795397 has a strong influence on promoter activity in vitro, in LCLs, its effect on *ZPBP2* transcription is masked by DNA methylation of exon 1 of the *ZPBP2* gene. Moreover, DNA methylation levels of the *ZPBP2* exon 1 seem to depend upon the *cis*-regulatory haplotype as only LCLs that are homozygous for the HapB haplotype have lower exon 1 methylation and higher *ZPBP2* RNA levels (Fig. 3).

In summary, our data show that most allele-specific regulatory effects such as nucleosome occupancy, DNA methylation, and in vitro promoter and enhancer activity localize in a 5.3-kb region overlapping with the *ZPBP2* gene at least 31 kb away from the *ORMDL3* gene that shows allelic differences in expression [(Verlaan et al. 2009a) and this work]. The sum of our data suggests that this region harbors a strong enhancer. Our conclusions are also consistent with the Chromatin State Segmentation by HMM mapping results (http://genome.ucsc.edu/EncodeBroadHmm) (Ernst and Kellis 2010; Ernst et al. 2010). It remains to be determined if the *ZPBP2* enhancer region exerts a long-range regulatory effect that extends beyond the *ZPBP2* gene and contributes to the allele-specific differences in the expression of *ORMDL3* and other genes in the region (Verlaan et al. 2009a).

The functional SNP rs4795397 is located within the promoter region of *ZPBP2*, a gene whose importance for fertilization and male fertility has been demonstrated in both mice and humans (Lin et al. 2007; Redgrove et al. 2011). The rs4795397-A allele that boosts the *ZPBP2* promoter activity in vitro is also part of the asthma-associated haplotype HapA. The exon 1 of *ZPBP2* is unmethylated in human sperm (S. Berlivet and A. Naumova, unpublished) and cannot block the allelic effect of rs4795397 on gene expression. Therefore, it is conceivable that spermatozoa from male carriers of the asthma-associated rs4795397-A allele have a higher supply of the ZPBP2 protein and potentially an increased fertilization capacity. This may provide a slight advantage at the population level and lead to an increased transmission of the asthma-associated haplotype from fathers to offspring.

Our results provide an example where inter-individual variation in DNA methylation acts as a modifier of genetic influences on gene expression and may interfere with genetic mapping of *cis*-regulatory polymorphisms by attenuating the genetic effect on transcription and thereby the significance of genetic association results as in the case of the *ZPBP2* gene. Based on our results, we speculate that promoters and first exons of genes that show genetic *cis*-effect on expression levels with genome-wide statistical significance are likely not methylated at all, or else may have allele-specific methylation, where the low expressing allele would have high promoter methylation and vice versa.

DNA methylation of promoter and enhancer elements varies with cell type and/or developmental stage (Eckhardt et al. 2006; Ghosh et al. 2010). Therefore, cell-type specific DNA methylation patterns have to be taken into account in the search for candidate disease gene. Several lines of evidence point to *ORMDL3* as the best 17q21 candidate causal gene for childhood asthma. Its expression levels show association with genotype in LCLs (Moffatt et al. 2007; Verlaan et al. 2009a, b), T lymphocytes (Murphy et al. 2010) and cord blood lymphocytes (Lluis et al. 2011). *ORMDL3* is also expressed in bronchial epithelial cells and its RNA levels are slightly higher in asthmatic subjects compared to controls, whereas *GSDMB* and *ZPBP2* are practically not expressed (Bochkov et al. 2010). Whether or not *ORMDL3* is the causal gene responsible for predisposition to asthma remains to be addressed using other approaches. It is important to note, however, that while the current experimental evidence excludes *IKZF3* (*IKZF3* is not affected by the haplotype effect in LCLs or T lymphocytes), it is not sufficient for ruling out *ZPBP2, GSDMB* or *GSDMA* as contributors to predisposition to asthma. Allelic transcription of *GSDMB* in LCLs and T lymphocytes has been previously demonstrated (Verlaan et al. 2009a, b; Murphy et al. 2010). As for *ZPBP2* and *GSDMA,* it is possible that in certain cell types their promoters may be unmethylated and their transcription may also depend on the haplotype. To exclude *ZPBP2*, *GSDMB* and *GSDMA* and narrow down the list of candidate genes for predisposition to asthma expression studies in cell types that are relevant for the etiology of asthma are necessary.

## Electronic supplementary material

Below is the link to the electronic supplementary material.

Supplementary material 1 (DOC 201 kb)
