# Supplementary material for: Interaction between genetic and epigenetic variation defines gene expression patterns at the asthma-associated locus 17q12-q21 in lymphoblastoid cell lines
Source: Hum Genet. 2012 Jan 24;131(7):1161–71. doi: 10.1007/s00439-012-1142-x (PMC3374122; doi:10.1007/s00439-012-1142-x)

**SUPPLEMENTARY MATHERIALS**

**FOR**

INTERACTION BETWEEN GENETIC AND EPIGENETIC VARIATION DEFINES GENE EXPRESSION PATTERNS AT THE ASTHMA-ASSOCIATED LOCUS 17q12-q21 IN LYMPHOBLASTOID CELL LINES.

Soizik Berlivet, Sanny Moussette, Manon Ouimet, Dominique J Verlaan, Vonda Koka, Abeer Al Tuwaijri, Tony Kwan, Daniel Sinnett, Tomi Pastinen, Anna K.Naumova

*For certain assays several sets of primers were used to determine optimal combinations. In those cases when the results obtained with several sets of primers were of a similar quality (the rs4795397 region and *GSDMB* promoter), they were combined and included in the paper.

**Supplementary Figure 1S**: Selection of putative enhancer regions with allelic effects based on ENCODE data.

Caption of the ENCODE Integrated Regulation Tracks (<http://genome.ucsc.edu/cgi-bin/hgTrackUi?hgsid=215814559&c=chr17&g=wgEncodeReg>) data for H3K4me1, H3K27Ac, H3K4me3, NFκB, POL2 and EP300 enrichment. These data were used to select the putative enhancer regions that were tested in *in vitro* assays for enhancer activity.

The cis-regulatory haplotype region, associated SNPs, the regions tested *in vitro* for promoter and enhancer activity are indicated. The functional SNP rs12936231 and rs4795397 are shown in red. The promoter regions that show highly significant (P<0.01) allelic differences *in vitro* are also shown in red.


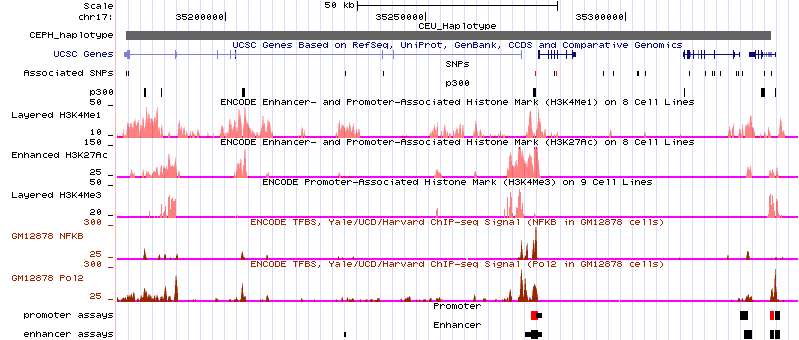


**Supplementary Figure 2S** *ORMDL3* promoter methylation does not depend upon haplotype in LCLs. Filled circles represent methylated cytosines, open circles represent unmethylated cytosines in CG pairs. Each row represents the methylation pattern of a single clone, i.e. one allele. The CG ID number is shown on the top of the panel below the ID of the LCL. The number of clones with the same methylation profile is shown on the right. The haplotype is shown on the top left.


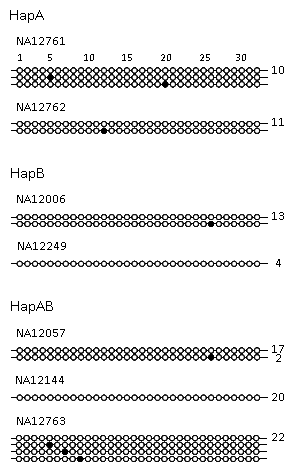


**Supplementary Figure 3S** DNA methylation profile of the *IKZF3* promoter does not depend upon the haplotype. *IKZF3* promoter methylation in LCLs that are homozygous for either the HapA or the HapB haplotype. The region harbors SNP rs1453559 with the A allele corresponding to the HapA and rs1453559-G allele corresponding to the HapB haplotype. Open circles represent unmethylated CG sites, filled black circles represent methylated CG sites and gray circles represent non-informative CGs. Numbers above the circles indicate the CG ID number. Each row represents an individual clone. The number shown on the right reflects the number of clones with same methylation pattern. The DNA sample ID is shown on the top.


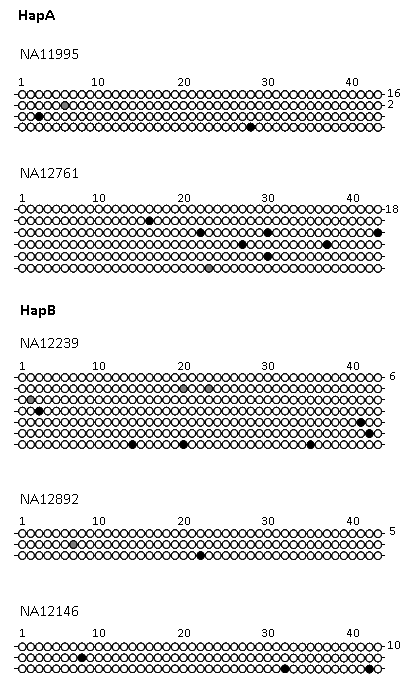


**Supplementary Figure 4S** *GSDMB* promoter is highly methylated in LCLs. Filled circles represent methylated cytosines, open circles represent unmethylated cytosines in CG pairs. Each row represents the methylation pattern of a single clone, i.e. one allele. The CG ID number is shown on the top of the panel below the ID of the LCLs. The number of clones with the same methylation profile is shown on the right. The haplotype of the LCL is shown on top left. The *GSDMB* promoter region harbors SNPs rs9303281 and rs9303280. Rs9303281 abolishes the CG#3 and an ins/del polymorphism abolishes CG #6 and 7 sites on the HapA allele.


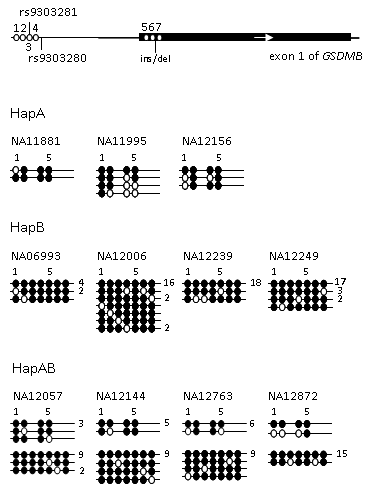


**Supplementary Figure 5S**. DNA methylation profiles of the *GSDMA* promoter region vary among LCLs. The analyzed region harbors SNP rs3902025 with the A allele corresponding to the HapA and the C allele corresponding to the HapB haplotype. The DNA sample ID is shown on the top. The haplotype of the LCL is shown above the sample ID. Open circles represent unmethylated CG sites and filled circles represent methylated CG sites. Each row represents an individual clone. The number shown on the right reflects the number of clones with same methylation pattern.


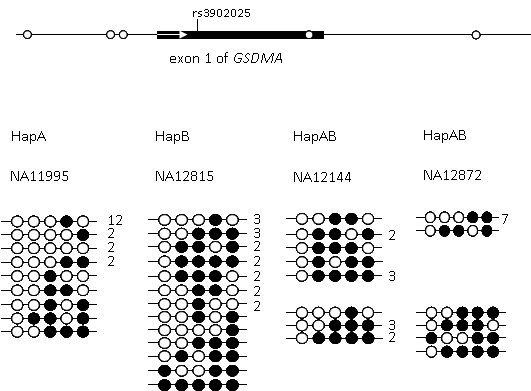


**Supplementary Figure 6S** The proximal *ZPBP2* promoter region that harbors functional SNP rs4795397 is unmethylated in LCLs independent of haplotype. Open circles represent unmethylated CG sites and filled circles represent methylated CG sites. The numbers above the circles show the CG ID number. Each row represents an individual clone. The number of clones with the same methylation pattern is shown on the right. The DNA sample ID is shown above the methylation diagram. The haplotype of the LCL is shown on the top.


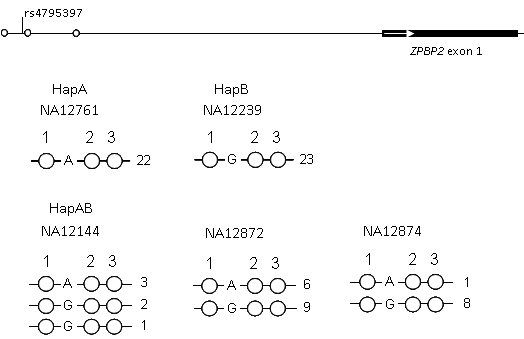

Supplement: Supplementary file 1 — Supplementary material 1 (DOC 201 kb) [file 439_2012_1142_MOESM1_ESM.doc]
